# Genome-Guided Analysis of Physiological Capacities of *Tepidanaerobacter acetatoxydans* Provides Insights into Environmental Adaptations and Syntrophic Acetate Oxidation

**DOI:** 10.1371/journal.pone.0121237

**Published:** 2015-03-26

**Authors:** Bettina Müller, Shahid Manzoor, Adnan Niazi, Erik Bongcam-Rudloff, Anna Schnürer

**Affiliations:** 1 Department of Microbiology, Swedish University of Agricultural Sciences, BioCenter, Uppsala, Sweden; 2 Department of Animal Breeding and Genetics Science, Swedish University of Agricultural Science, SLU-Global Bioinformatics Centre, Uppsala, Sweden; 3 University of the Punjab, Lahore, Pakistan; Louisiana State University and A & M College, UNITED STATES

## Abstract

This paper describes the genome-based analysis of *Tepidanaerobacter acetatoxydans* strain Re1, a syntrophic acetate-oxidising bacterium (SAOB). Principal issues such as environmental adaptations, metabolic capacities, and energy conserving systems have been investigated and the potential consequences for syntrophic acetate oxidation discussed. Briefly, in pure culture, *T*. *acetatoxydans* grows with different organic compounds and produces acetate as the main product. In a syntrophic consortium with a hydrogenotrophic methanogen, it can also reverse its metabolism and instead convert acetate to formate/H_2_ and CO_2_. It can only proceed if the product formed is continuously removed. This process generates a very small amount of energy that is scarcely enough for growth, which makes this particular syntrophy of special interest. As a crucial member of the biogas-producing community in ammonium-rich engineered AD processes, genomic features conferring ammonium resistance, bacterial defense, oxygen and temperature tolerance were found, as well as attributes related to biofilm formation and flocculation. It is likely that *T*. *acetatoxydans* can form an electrochemical gradient by putative electron-bifurcating Rnf complex and [Fe-Fe] hydrogenases, as observed in other acetogens. However, genomic deficiencies related to acetogenic metabolism and anaerobic respiration were discovered, such as the lack of formate dehydrogenase and F_1_F_0_ ATP synthase. This has potential consequences for the metabolic pathways used under SAO and non-SAO conditions. The two complete sets of bacteriophage genomes, which were found to be encoded in the genome, are also worthy of mention.

## Introduction

In anoxic habitats where inorganic electron acceptors such as nitrate, manganese, iron or sulphate are absent, organic matter degradation proceeds dominantly through methanogenesis [1]. Methane of biological origin is generally produced by methanogenic archaea from either acetate, hydrogen or methyl group-containing substrates [2]. While hydrogen is a more energetically favourable substrate, acetate is usually the quantitatively more available substrate, being a central intermediate during the anaerobic degradation of different organic compounds [1, 3]. Methane formation from acetate can proceed through two different pathways: 1) direct cleavage of acetate by aceticlastic methanogens [4, 5] and 2) syntrophic acetate oxidation (SAO) [6, 7]. The latter pathway involves two sets of reactions whereby acetate is first converted to H_2_ and CO_2_ by acetate-oxidising bacteria (SAOB). In a second step, involving a hydrogenotrophic methanogen, CO_2_ is reduced to methane. For thermodynamic reasons, methane formation via SAO can only proceed at low partial pressures of hydrogen and in a strictly syntrophic relationship between the organisms involved [8, 9].

SAO has been observed in a number of natural and artificial anoxic environments such as rice paddyfield, soil and subtropical lake sediments [10–12], oil reservoirs [13], nutrient-enriched soils [14] and biogas digesters [7, 15–19]. SAO is energetically less favourable than aceticlastic methanogenesis, as two organisms have to share a very small amount of energy that is hardly enough for one [9, 20]. Nevertheless, SAO occurs in natural environments, usually dominated by methanogens. At present the parameters that regulate the competition between the two pathways are not fully known, but some factors suggested to be of importance are ammonia level, acetate concentration, temperature, aceticlastic community structure and dilution rate [15, 16, 19, 21, 22].

To date, three mesophilic SAOB, namely *Clostridium ultunense*. [23, 24], *Syntrophaceticus schinkii* [24], and *Tepidanaerobacter acetatoxydans* [25], and two thermophilic SAOB, namely *Thermacetogenium phaeum* [26] and *Thermotoga lettingae* [27], have been isolated and characterised. All these SAOB were originally isolated from different anaerobic reactors and all but one are affiliated within the phylum Firmicute*s* to the *Clostridia* class. *T*. *lettingae* belongs to the phylum Thermotogae. In pure culture, these bacteria have the ability to use different organic substrates such as carboxylic acids, amino acids and alcohols and produce acetate as their main product. In addition, *T*. *phaeum* and *T*. *lettingae* can grow autotrophically using hydrogen/carbon dioxide as substrate [26, 27]. The number of substrates used is restricted for *S*. *schinkii*, *C*. *ultunese* and *T*. *phaeum*, but appears to be rather broad for *T*. *acetatoxydans* and *T*. *lettingae*. A feature in common for the mesophilic SAOB is ammonia tolerance, with *T*. *acetatoxydans* being the most robust SAOB. Another typical feature of this organism is a broad temperature range (25–55°C), with an optimum between the mesophilic and thermophilic range at 44–45°C.

Among this limited number of isolated SAOB, two complete genome sequences, of *T*. *phaeum* [28] and *T*. *acetatoxydans* [29], and one draft genome sequence, of *C*. *ultunense* [30], have been published. However, so far only the genome of the thermophilic *T*. *phaeum* has been more thoroughly analysed [28]. The aim of the present study was therefore to gain further information about the SAOB regarding their potential physiological and morphological traits by performing a genome-scale analyse of the first complete genome sequence of a mesophilic SAOB. In the analysis, general genome features were characterised and issues relating to environmental adaptation, substrate utilisation capacity, energy conservation and syntrophic acetate oxidation were examined.

## Results and Discussion

### General genome features

The major features of the *T*. *acetatoxydans* genome are listed in Table 1 and are summarised in Manzoor *et al*. [29]. The *T*. *acetatoxydans* genome contains 2,656 predicted protein-coding sequences (CDS), of which 2,053 (77.29%) were assigned tentative functions, 603 predicted proteins (21.27%) matched proteins of unknown function and the remaining 38 (1.4%) did not give any database matches. 2,158 (81.25%) CDS could be allocated to the 21 functional COGs (Cluster of Orthologous Groups), to the same range as found for other sequenced acetogenic bacteria such as *Acetobacterium woodii* and *Moorella thermoacetica*. The largest numbers of genes fall into four main categories: amino acid transport and metabolism, energy metabolism, carbohydrate transport and metabolism, and inorganic ion transport and metabolism (S1 Table).

**Table 1:** General genomic features of *Tepidanaerobacter acetatoxydans* strain Re1.

Genome size (base pairs)	2,761,252
G+C content	37.5
Open reading frames	2852
Predicted protein-encoding sequences	2656
Intergenic length (bp)	136.12
Coding density	86.92
rRNA	6
tRNA	52
Genes with function prediction (percentage)	2102 (77.82%)
Number of genes in COG (percentage)	2148 (79.52%)
Genes encoding signal peptides (percentage)	179 (6.73)
Genes encoding transmembrane proteins (percentage)	628 (23.64)
Gene remnants	5

The MaGe annotation server identified five gene remnants; two of them are redundant genes having paralogues within the genome. *T*. *acetatoxydans* belongs to the Firmicutes-Clostridia class [25] and its closest relatives are members of the genera *Thermovenabulum*, *Tepidanaerobacter* and *Thermosediminibacter*. Synteny analysis with all available genomes in the NCBI RefSeq database (2014-03-21) confirmed the closest phylogenetic relationship inferred by 16S rRNA analysis to *Tepidanaerobacter syntrophicus*. However, *T*. *acetatoxydans* shows the maximum number of orthologues (1,294 or 48.72%) to *Thermosediminibacter oceani*, an anaerobic thermophilic bacterium isolated from marine sediment [31]. Comparing these, they appear to share some metabolic traits such as acetate forming sugar utilization, restricted usage of thiosulphate as terminal electron acceptor and inability to grow lithochemoautotrophically on H_2_/CO_2_ [25, 31].

#### Bacterial defence

The cas/CRISPR (Clustered Regularly Interspaced Short Palindromic Repeats) system is a prokaryotic defence mechanism widespread in bacteria and archaea [32]. It provides immunity against invasive DNA originating from *e*.*g*. phage attacks and plasmids by cleavage of single-strand DNA and RNA. The genome of *T*. *acetatoxydans* Re1 harbours two putative operons encoding *cas* proteins, including (TepRe1_0109 –_0115), two metal-dependent nuclease genes (*cas1* and *cas2*), along with a HD-nuclease (*cas3*) and an endonuclease gene (*cas6*). The second operon (TepiRe1_0129 – TepiRe1_0134) encodes proteins of the RAMP (Repeat-Associated Mysterious Proteins) superfamily. These CRISPR-associated sequence (*cas*) genes are often directly located adjacent to CRISPR loci [33], as is the case for *T*. *acetatoxydans*. Comparing all CRISPR-containing bacteria found in the database, the occurrence of CRISPR loci ranges from one to 21 and only 28 of these bacteria (4%), including *T*. *acetatoxydans*, harbour ten or more CRISPR loci. Engineered anaerobic digestion (AD) processes represent a habitat where microorganisms might be frequently exposed to phage attacks due to the feedstocks used (manure, municipal organic wastes *etc*.) and sequential enrichment due to the commonly used semi-continuous feeding approach. Little information is available in the literature about phage outbursts and phage enrichment in biogas processes and the consequences for process stability and community structure. Moreover, it is clear that a variety of different types of phages are produced in methanogenic digesters [34], while phages have also been identified in anaerobic reactors fed solely with acetate [35]. In line with this, we found three regions in the genome of *T*. *acetatoxydans* harbouring pro-phage-related genes, which ranged in size from 25 to 38 kb, representing a total of 3.57% (98.7 kb) of the genome. Two of these regions are predicted to be complete phages (S2 Table). Likewise, the SAOB *T*. *phaeum* isolated from a thermophilic methanogenic reactor has been shown to harbour an active prophage and a high number of CRISP-associated elements [28].

The acquisition of the CRISPR loci indicates adaptation to the species-rich AD environment triggered by continuous phage attacks and other transformation events.

#### Transport systems

179 genes were predicted to encode surface associated or secreted proteins, which seem to be exclusively translocated by the Sec system (TepRe1_0208, _0627–0629, _0818, _1182–1183, _1418–1419, _1969). Neither genes related to the TAT (twin-arginine translocation) mediated protein secretion pathway nor CDS encoding an N-terminal twin-arginine motif could be identified. 628 CDS are predicted to encode proteins having at least one transmembrane helix, including a minimum of 28 putative ATP-binding cassette (ABC) transport systems (S3 Table), six tripartite ATP-independent transporters (TRAP) (S4 Table), twelve secondary sodium/solute transporters (S5 and S6 Tables) and 15 putative phosphotransferase systems (PTS) (S7 Table). The six TRAP transporters might be considered an adaptation to the syntrophic lifestyle by this organism due to the following attributes reviewed by Kelly and Thomas [36]: i) An ion gradient is used for energising solute passage over the membrane, which is especially beneficial if the ATP reserves are strongly limited, as is the case under acetate-oxidising conditions; however ii) the transport is uni-directional, which is advantageous when maintaining homeostasis under the high osmotic pressure to which the organism is exposed. In comparison, its physiological relatives *M*. *thermoacetica* and *A*. *woodii* harbour only one and no TRAP, respectively.

### Environmental adaptations

The occurrence of *T*. *acetatoxydans* seems to be restricted to engineered AD processes. To date, no genotypes related to *T*. *acetatoxydans* have been detected in any other habitats (NCBI blastn search using either 16S rRNA (> 98% identity) or *fhs* sequence (> 96% nucleotide identity), 2014-07-25). Furthermore, the abundance of *T*. *acetatoxydans* in biogas processes is strongly correlated with increasing ammonium concentration [19, 22, 37]. The following section considers morphological and physiological traits supporting adaptation to the environmental conditions prevailing in AD processes digesting protein-rich feed.

#### Oxygen tolerance

Small amounts of oxygen continually enter the semi-continuously fed digestion process. Thus, micro-aero-tolerance and adaptation to unstable redox conditions is an advantage and gives increased competitiveness. Aero-tolerance has already been shown for other acetogens such as *M*. *thermoaceticum*, *Thermoanaerobacter kivui* and *Clostridium glycolicum* [38, 39], which express peroxidase and NADH oxidoreductase activity. However, *T*. *acetatoxydans* may express an alternative oxygen stress defence system consisting of two manganese-containing catalase genes (TepiRe1_0143, TepiRe1_2025), two rubrerythrin-encoding genes (TepiRe1_0311, TepRe1_1181), and two rubredoxin-encoding genes (TepiRe1_0396, TepiRe1_0397), enabling detoxification of superoxide and hydrogen peroxide. Manganese catalases are widely distributed non-heme catalases protecting organisms from peroxide stress [40]. Rubredoxin and rubrerythrin are non-heme iron proteins reported to be used as alternatives to superoxide dismutase and catalase by sulphate reducers [41].

#### Sporulation and chemotaxis

**Fig 1:**
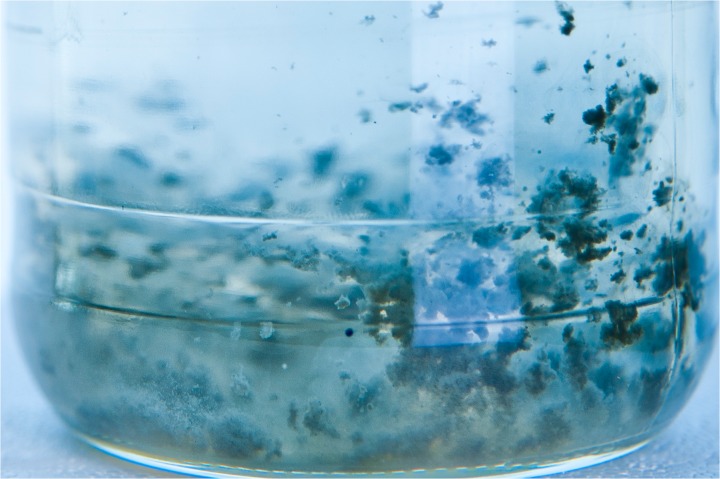
Methane production and growth of *Tepidanaerobacter acetatoxydans* and *Methanoculleus* spec. MAB2. A triplicate of co-cultures consisting of SAOB and the methanogenic partner *Methanoculleus* spec. MAB2 were cultivated on 100 mM acetate and SAO activity was followed by methane production, as described in the “Material and methods” section. Growth was monitored by quantification of the 16S rRNA gene by PCR in one of the cultures as described in the same section (The values obtained for *T*. *acetatoxydans* were divided by two). White square: *T*. *acetatoxydans*; white diamond: *Methanoculleus* spec. MAB2; filled triangle: methane production without acetate (Negative control); black square: methane production in the corresponding co-culture.

Nutritional starvation can be considered negligible in engineered biogas processes. Nevertheless, *T*. *acetatoxydans* is able to remain in a static phase for several weeks (Fig. 1). In addition, this bacterium appeared to be able to sporulate [25] as well as possesses sporulation genes, enabling it to outlast unfavourable environmental conditions and to colonise new habitats. As typically found in many Clostridia, the genome encodes the master regulator Spo0A (TepiRe1_1496) and lacks the histidine kinases Spo0F and Spo0B reviewed by Paredes-Sabja et al. [42]. All the sporulation-specific sigma factors SigE (TepiRe1_1251), SigG (TepiRe1_1252), SigF (TepiRe1_1488), and SigK (TepiRe1_1533) are also predicted.

**Fig 2:**
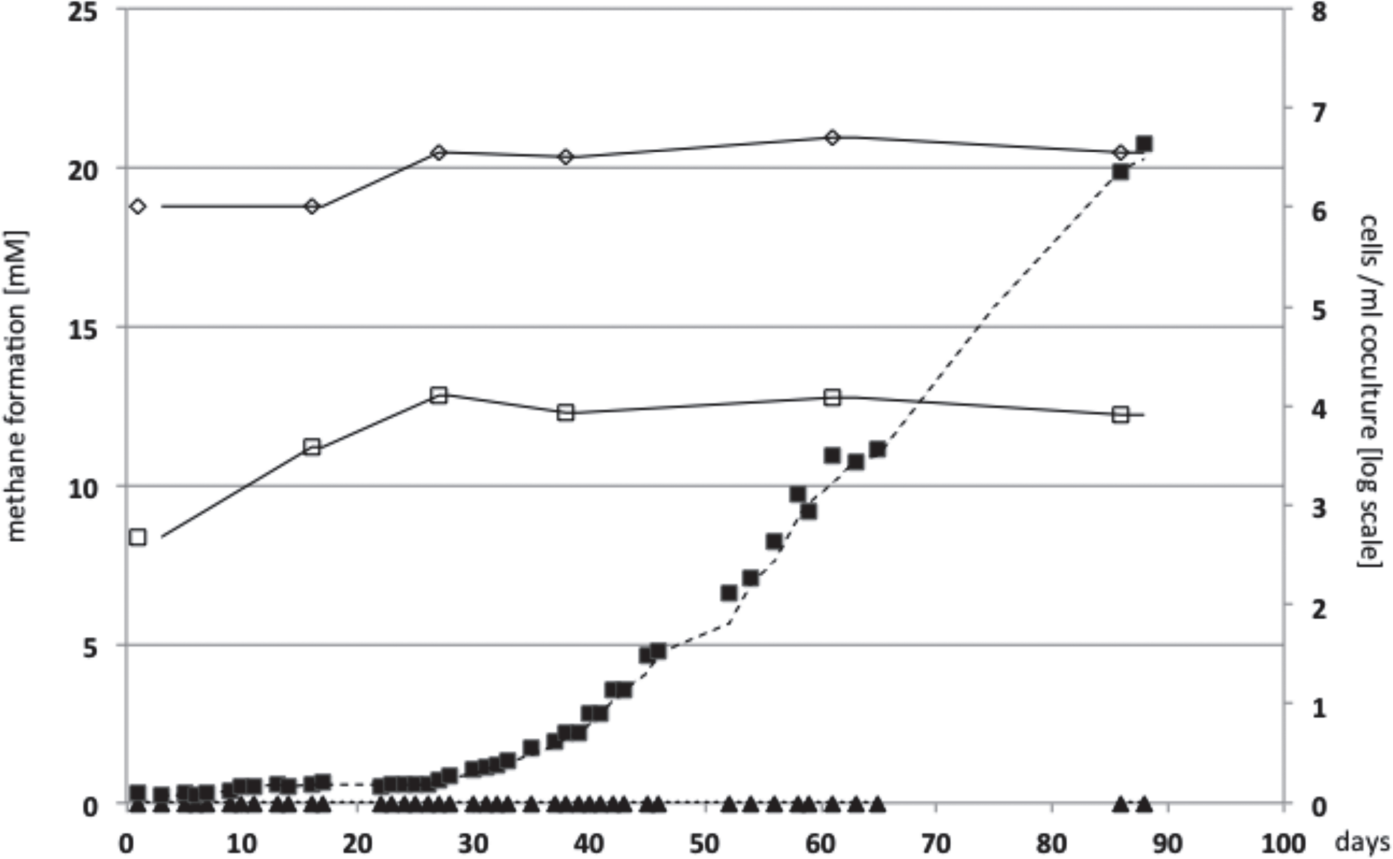
Acetate oxidizing co-culture consisting of SAOB and *Methanoculleus* species MAB2 cultivated, as described in the “Material and methods” section (flocs are between 2 mm and 5 mm in diameter).

Furthermore, *T*. *acetatoxydans* has been shown to express a single polar flagellum during the early exponential growth phase, enabling spinning movements [25]. Motility and chemotactic attributes improve nutrient supply, increase the likelihood of interactions between the bacterium and its methanogenic partner and may also promote surface adhesion. A mixed co-culture containing *T*. *acetatoxydans* and the methanogenic partner was shown to grow in dense flocs, possibly formed by active movement (Fig. 2). Explaining the observed motility, the genome contains 42 flagellum-associated genes organised in three operons. Flagella class III proteins, flagella formation proteins, motor proteins, basal proteins and biosynthesis secretory proteins are encoded in the first operon (TepiRe1_1326–1361). The second operon (TepiRe1_0891–0899) harbours genes involved in flagellum assembly, *i*.*e*. hook-associated proteins and flagella biosynthesis anti-sigma factor protein, while capping protein are encoded in the third operon (TepiRe1_0923–0928). Genes encoding the accessory flagella assembly proteins FlgA, FlhC, FlhD and FliT could not be identified. The first operon also encodes the basic chemotaxis machinery necessary for regulation of flagella-based movement [43]. This consists of a putative signal transduction histidine kinase CheA (TepiRe1_1355), a response regulator CheY (TepiRe1_1345) and the scaffolding protein CheW (TepiRe1_1356). Potential homologues to CheR (TepiRe1_1451) and CheB (TepiRe1_1267), which are needed for fine-tuning, were also found, enabling *T*. *acetatoxydans* to sense wide ranges of nutrient concentrations.

#### Flocculation

The participation of the flagellum and the flagellum apparatus in surface adherence and observed floc formation (Fig. 2) might be highly important for *T*. *acetatoxydans* in avoiding wash-out in the continuous biogas process by forming microniches and establishing syntrophic interactions. Even though little information is available in the literature, chemotaxis-like signal transduction cascades have been found to be involved in biofilm formation and flocculation [43]. Flocs and biofilm formation involve the assembly of non-motile cells in an exopolysaccharide (EPS) matrix. During the late exponential growth phase, *T*. *acetatoxydans* loses its motility and instead forms long chains indicating presence of EPS [25]. It has been hypothesised for the soil bacterium *Azospirillum brasilense* that the EPS production is Che-regulated and promotes clumping and flocculation [44,45]. Likewise, biofilm formation by *Pseudomonas* species employs the chemotaxis proteins [46]. The chemosensoric systems employed for those functions consist of the core structure similar to the one found in *T*. *acetatoxydans* and described in section “Sporulation and chemotaxis” and additional components, only a few of which have been analysed [43].

Moreover, *T*. *acetatoxydans* harbours a cluster encoding potential type IV pili-related proteins (TepiRe1_0678–0690), including a prepelin peptidase, a hexameric ATPase, pilus assembly proteins and proteins related to the Tad (tight adherence) macromolecular transport system. Type IV pili are retractable and are needed for transformation and phage transduction, both important mechanisms for the occurrence of lateral gene transfer (reviewed in [47]). Type IV pili are also involved in biofilm formation and may inherit the same function in flocculation. Here the retractable attribute might be important to bring the syntrophic partners in close proximity to each other in order to guarantee the low hydrogen partial pressure making overall acetate oxidation thermodynamically favourable. Considering the low amount of energy available, which needs to be shared by the syntrophic partners, the type IV pili might be even used by *T*. *acetatoxydans* for exchanging H_2_ or formate or for direct electron transfer, as reviewed by Shrestha and Rotaru [48]. A putative retraction ATPase gene is predicted elsewhere in the genome (TepiRe1_2344).

A second predicted operon (TepiRe1_1523–1531) harbouring pili-related genes was identified, but might be assignable to competence rather than to pilus formation due to a predicted late competence development protein. Furthermore, no small-sized proteins as typically described for pilin subunits are encoded by this operon. Thus, this operon might instead express the uptake machinery needed for transformation.

#### Homeostasis and ammonium tolerance

One of the most interesting features of *T*. *acetatoxydans* is its adaptation to the high-osmolality conditions, especially the high ammonium levels, prevailing in AD processes digesting protein-rich feed. It can easily cope with ammonium concentrations up to 1 M and has been shown to benefit from increasing ammonium levels by outcompeting aceticlastic methanogens, which in this regard lack effective osmoprotective ability. This enables the expansion of SAO to the extent observed in high ammonium processes [15, 19, 22, 37, 49]. The initial rapid uptake of potassium ions is known to be the first adaptive response by bacteria to high osmolarity, followed by accumulation of compatible solute [50]. We found two potential K+ uptake transporters (TepiRe1_0362–0363; TepiRe1_0730–0737) encoded in the *T*. *acetatoxydans* genome. In a second response, *T*. *acetatoxydans* might accumulate and/or synthesise a large amount of metabolically inert osmolytes. For example, glutamate, proline and betaine have been described as frequently used osmoprotectants in Gram-positive bacteria [50]. At least one of the 28 encoded ABC transport systems (S3 Table) in *T*. *acetatoxydans* was predicted to transport proline, betaine and glycine, but more may be involved, since the model organism *B*. *subtilis* expresses at least five different primary and secondary transport machineries with different substrate spectra and affinities [50]. Here, TRAP transporters might be employed rather than ABC or secondary transporters due to the advantages described above. In addition, *T*. *acetatoxydans* harbours genes needed for *de novo* synthesis of L-proline from glutamate (TepiRe1_0035, 2283, 2284) or from ornithine (TepiRe1_2520). We found a few more interesting features in the *T*. *acetatoxydans* genome that might be specifically related to ammonia tolerance: At first no ammonium transporters were predicted, although these are usually expressed under ammonium limited conditions [51]. In comparison, the genomes of the acetogens *M*. *thermoacetica* and *A*. *woodii* encode several homologues each. The risk of leakiness is therefore strongly reduced in *T*. *acetatoxydans* and the charged ammonium ion does not readily pass over the cytoplasm membrane itself. However, ammonium is in equilibrium with ammonia, which does pass over the membrane. Inside the cell, the same equilibrium raises the pH, affecting enzyme activity. At neutral pH, the proportion of ammonia (*pK*_*s*_ 9.0) would be low and probably not toxic. However, the continuous release of ammonium in AD processes digesting protein-rich feed results in pH values of around 8.0, with the consequence of a higher proportion of ammonia. This proportion increases further when the temperature increases. One adaptation to the influx of ammonia might be the absence of a potential glutamine synthetase gene, to avoid depleting the ATP reserves of the cell, which is usually expressed as part of the GS/GOGAT ammonium assimilation mechanism in prokaryotes. Instead, we found a potential glutamate dehydrogenase gene (TepiRe1_1416) located together with 2-ketoglutarate synthase-encoding genes (TepiRe1_1413–1414), forming 2-ketogluterate as one of the substrates needed by glutamate dehydrogenase. This enzyme has a low affinity for ammonium, is poorly regulated and does not function at the expense of ATP. Thus it might have the function of scavenging ammonia from the cytosol, while at the same time glutamate is formed for biosynthesis. Since it harbours the gene set needed for proline formation from glutamate, ammonia scavenging might be directly linked to osmolyte production in order to avoid water efflux and maintain turgor under high ammonium pressure. Moreover, *T*. *acetatoxydans* appears to depend on amino acid rich environments, which might support a glutamate dehydrogenase function in detoxification rather than in ammonium assimilation. Glutamate synthase activity (encoded by TepiRe1_0414) might also play a role in detoxification, because of the reversibility of the ammonia-dependent reaction [52]. Furthermore, four potential Na+/H+ antiporters (TepiRe1_0140, _0933, _1094, 2020, 2198) and two V-type ATP synthases (S5 Table; see section on energy conservation) might support the organism in maintaining pH homeostasis. These adaptations might explain why *T*. *acetatoxydans* has not yet been discovered in habitats where the availability of ammonium is limited and/or the pH does not favour ammonia formation.

#### Temperature tolerance

Temperature fluctuations, but also changes in intended process temperature, commonly prevail in AD processes. *T*. *acetatoxydans* has been shown to be adapted to temperatures between 20°C and 55°C [19, 30]. In order to stabilise, fold and refold proteins, heat shock proteins Hsp and other proteins related to temperature adaptation might be important for survival in this habitat. In total, we found ten genes encoding potential heat shock proteins. Hsp40 (DnaJ) gene and Hsp70 (DnaK) gene were arranged in a cluster together with the genes for Hsp70 co-chaperone GrpE, chaperonin GroEL and a heat-inducible transcription repressor (TepiRe1_1194-_1198). Genes encoding Hsp 100 (ClpB: TepiRe1_1926), a Clp protease (TepiRe1_0661–0662), two Hsp20 homologues (TepiRe1_0528, 2322) and GroES/GroL (TepiRe1_0435–0436) were scattered in the genome. Growth at lower temperatures has been observed, so a putative cold shock protein Csp might be expressed under such temperature shifts (TepiRe1_0874).

### Substrate utilisation

*T*. *acetatoxydans* appears to have CcpA-dependent regulation of its carbon metabolism, as usually observed for Gram-positive bacteria: HPr kinase/phosphatase gene (TepiRe1_0749) and an HPr homologue (TepiRe1_0766) have been predicted. Multiple sequence alignment proved the existence of both phosphorylation sites, the so-called regulatory site at Ser46 and the so-called metabolic site at His15 in HPr, suggesting tight regulation of carbon metabolism both catalytically and regulatory. TepiRe1_1974, _0704, _0777, _1955 and _2214 encode homologues to the transcriptional regulator CcpA. The phosphotransferase system enzyme I (TepiRe1_1088) is encoded elsewhere.

In line with its ability to ferment sugars and sugar derivatives such as glucose, fructose, mannose, lactose, cellobiose, salicin and glycerol [30], the genome encodes all the enzymes needed for the Embden-Meyerhof-Parnas (EMP) pathway, organised in three clusters. The first cluster encodes enolase (TepiRe1_2125), phosphoglycerate mutase (TepiRe1_2126), triosephosphate isomerase (TepiRe1_2127), phosphoglycerate kinase (TepiRe1_2128) and glyceraldehyde-3-phosphate dehydrogenase (TepiRe1_2130). Hexokinase (TepiRe1_0803–0804), fructose-1,6-bisphosphate aldolase (TepiRe1_0805) and glucose phosphate isomerase gene (TepiRe1_0806) are located together in a second cluster. The third cluster harbours genes for 6-phosphofructokinase (TepiRe1_0770) and pyruvate kinase (TepiRe1_0771). Several predicted glucose-, mannose- and fructose-specific phosphoenol pyruvate-dependent phosphotransferase systems (PEP-PTS) were found (S7 Table). Fructose-6-phosphate can enter the EMP pathway as fructose-1,6-phosphate by the activity of 1-phosphofructokinase (TepiRe1_0807), whereas mannose-6-phosphate is probably converted to fructose-6-phosphate by the predicted bi-functionality of the glucose phosphate isomerase identified in the first cluster. Five 6-phospho-β-glucosidases (TepiRe1_0055, 0276, 1754, 2361, 2425) hydrolysing phosphocellobiose to glucose and glucose-6-phosphate were found, two of which (TepiRe1_2357–2359; TepiRe1_2427–2429) cluster together with predicted cellobiose-specific PTS (S7 Table). The toxic phenol glycoside salicin, a secondary plant chemical, might be metabolised in the same way. Since no salicyl aldehyde-degrading enzymes were predicted, only the glycoside moiety seems to be used by the cells. Lactose might be taken up and phosphorylated by PTS (S7 Table), and is subsequently hydrolysed to glucose and galactose-6-phosphate by β-galactosidase activity (TepiRe1_0812). Since no enzyme except an UDP-phosphogalactose phosphotransferase (TepiRe1_1916) could be found and *T*. *acetatoxydans* has been clearly shown to ferment free galactose, galactose-6-phosphate might be further metabolised to glycerinaldehyde-3-phosphate and dihydroxyacetone phosphate by the D-tagatose-6-phosphate pathway. The presence of genes predicted to encode putative tagatose-6-phosphate isomerases (TepiRe1_2386, 2274), kinase (TepiRe1_0807) and aldolase (TepiRe1_0298) supports the existence of that pathway. Glycerol most likely crosses the membrane by passive diffusion, because no predicted glycerol facilitator or aquaglyceroporins were found, although two genes (TepiRe1_0552, 0621) encoding the glycerine uptake regulatory protein GlpP [53] were predicted. It probably enters the EMP pathway at the level of dihydroxyacetone phosphate, most likely oxidised by the activity of glycerol kinase (TepiRe1_0624, 2305, 2381) and an FAD-dependent glycerol-3-phosphate dehydrogenase (TepiRe1_1262). The genome of *T*. *acetatoxydans* appears to encode a complete reductive TCA cycle used by anaerobic bacteria to generate metabolic intermediates and to oxidise organic compounds such as citrate, malate and pyruvate, as seen for this organism [25]. A putative citrate synthase, aconitate hydratase and isocitrate dehydrogenase are encoded by ORF (TepiRe1_0166–0168). Malate dehydrogenase and fumarase form another cluster (TepiRe1_0545–0547). 2-ketoglutarate:ferredoxin oxidoreductase activity might be encoded by three of the predicted 2-oxoacide:ferredoxin oxidoreductases (TepiRe1_2397–2398; 0074–0075; 1413–1415). A cluster consisting of ORFs TepiRe_1962–1971 harbours potential homologues for succcinyl-CoA:acetate CoA transferase and fumarate reductase genes. Another locus encodes similar genes to the citrate lyase complex (TepiRe1_2443–2445). In addition, a putative pyruvate:ferredoxin oxidoreductase (TepiRe1_2143), pyruvate carboxylase (TepiRe1_1425), PEP carboxykinase (TepiRe1_0184) and glucose-6-phosphatase (TepiRe1_0750), as needed for gluconeogenesis, have been identified. However, fructose-1,6 bisphosphatase seems to be absent in *T*. *acetatoxydans* and is instead replaced by a pyrophosphate-dependent fructose-6-phosphate-1-transferase (TepiRe1_0526).

It has been shown that *T*. *acetatoxydans* can grow on lactate, but only when thiosulphate is present as a terminal electron acceptor, as also seen for its closest known relative *T*. *syntrophicus*, with which it shares 96% 16S rRNA identity [25]. In the case of *T*. *acetatoxydans*, a D-lactate dehydrogenase gene (TepiRe1–2534) was found, organised in an operon together with a predicted lactate permease (TepiRe1_2531) and two genes encoding a putative electron transferring flavoprotein ETF (TepiRe1_2532, 2543), which might shuffle the electrons via FADH to thiosulphate.

### Amino acid and coenzyme biosynthesis

*T*. *acetatoxydans* can grow without any supplementary amino acids, but at very reduced rates [25]. Accordingly, the ABC transport systems present are predicted to transport amino acids as well as peptides (S3 Table) amongst others. Ammonium assimilation might occur via low affinity glutamate dehydrogenase as mentioned above. However, using the metabolic profile options on MaGe, *T*. *aceatoxydans* still seems to be restricted in its ability to synthesise a few of the essential amino acids compared to the genome of other acetogens such as *M*. *thermoaceticum* and *A*. *woodii*. Alternative pathways may be used by *T*. *acetatoxydans* to synthesise the following amino acids, explaining the observed slow growth without supplementary amino acids: Asparagine might be produced by tRNA-dependent transamination and serine might be formed in a tetrahydrofolate-dependent reaction performed by glycine hydroxyl methyl transferase (TepiRe1_2273). Only one branched chain amino acid transaminase is predicted (TepiRe1_2082), which must therefore be employed by the L-isoleucine, L-leucine and L-valine biosynthesis pathways. Genes encoding key enzymes necessary for sulphate assimilation, such as ATP sulphurylase and APS kinase, are absent. Instead, *T*. *acetatoxydans* might incorporate the sulphur needed for synthesis of sulphur-containing amino acids, sulphur-containing coenzymes and prosthetic groups on the level of sulphide by the activities of serine O-acetyl transferase (TepiRe1_2341) and cysteine synthase (TepiRe1_0834). However, biosynthesis of arginine remains undiscovered. *T*. *acetatoxydans* seems not to express any selenocysteine-containing proteins; L-selenocysteinyl-tRNA^Sec^ synthase (TepRe1_1692) and selenophosphate synthase (TepRe1_0426) were predicted, but the selenocysteinyl-tRNA specific elongation factor SelB was not found in the genome.

In line with the requirements for nutrient supplements for laboratory cultivation, the genome of *T*. *acetatoxydans* lacks either parts or complete gene sets necessary for synthesis of coenzyme precursors such as cobalamine, nicotinic acid, folic acid, pyridoxine, thiamine, biotin and pantothenic acid derivatives. The anabolic restrictions described here are in line with the observed growth limitations of *T*. *acetatoxydans* and can be considered an adaptation to the AD environment. They also explain its rareness in nutrient-limited habitats.

### Acetogenesis

*T*. *acetatoxydans* has been described as an homoacetogen, producing acetate as the only end product using the Wood-Ljungdahl (WL) pathway when growing heterotrophically [25, 54]. In general, taking glucose as an example, three moles of acetate are produced from one mole of glucose, two of which are produced from pyruvate either by a pyruvate dehydrogenase multi-enzyme complex (TepiRe1_0698–0701) or by a pyruvate:ferredoxin oxidoreductase complex PFOR (TepiRe1_2368–2371) and the subsequent activity of phosphate acetyl transferase (TepiRe1_1559) and acetate kinase (TepiRe1_1558), regenerating a total of four moles of ATP net by substrate-level phosphorylation. The third mole of acetate is produced by anaerobic respiration employing the WL pathway to oxidise the reduction equivalents produced during the EMP pathway by reducing two moles of CO_2_ to acetyl-CoA. Acetyl CoA is then further converted to acetate by the activity of acetyl transferase and acetate kinase, without any net ATP synthesis. The WL pathway genes were found to be organised in one operon (TepiRe1_0611–0630), as already partly identified by [54], but no formate dehydrogenase function catalysing the first step in the CO_2_ reduction chain to a methyl group could be predicted, either within the operon or elsewhere in the genome. This finding basically confirms the observed inability of this organism to establish a chemoautotrophic lifestyle [25] and raises the question of how the third mole of acetate can be formed, especially as growth on formate has not been observed [25]. Under heterotrophic growing conditions, *T*. *acetatoxydans* might link the glycolytic pathway to the reductive WL pathway by employing a pyruvate formate lyase (TepiRe1_0046) rather than a pyruvate dehydrogenase or PFOR (see above), releasing acetyl CoA and formate instead of CO_2_. As a hypothetical consequence, only substrates passing through pyruvate formate lyase can most likely be used by this species. For example, genes encoding putative alcohol, aldehyde and lactate dehydrogenases (TepiRe1_0393, 0142, 2534) are predicted, but ethanol, butanol, propanol or lactate consumption has not been observed (except with thiosulphate as electron acceptor, see below) [25]. In contrast, the acetogen *M*. *thermoacetica* [55], which expresses formate dehydrogenase activity [56], can oxidise those substrates. Genes encoding enzymes belonging to the methyl branch of the WL pathway, such as methyl transferase, formyltetrahydrofolate synthetase, methenyltetrahydrofolate cyclohydrolase, methylenetetrahydrofolate dehydrogenase and methylenetetrahydrofolate reductase, exist as duplicates and were found organised in a cluster (TepiRe1_0337–0342), as reported by Müller et al. [54]. Based on mRNA expression studies, those authors suggested that the second cluster is an alternative set of genes required for the intermediate C1 carbon metabolism, when the WL operon becomes down-regulated. A third methenyltetrahydrofolate cyclohydolase gene is located elsewhere (TepiRe1_0842).

The acetate produced might be removed from the cytoplasm by a predicted formate/nitrite transporter (TepiRe1_2032). It has been shown for *Salmonella typhimurium* that the formate/nitrite transporter FocA translocates all products of the mixed acid fermentation apart from formate and nitrite, with an efficiency of 45% for acetate [57]. Due to the high degree of conserved residues forming the transport channel of this family’s members, it is suggested that substrate flexibility might be a general feature of all proteins belonging to the family [57].

### Energy conservation

Three genes encoding putative ferredoxins (TepiRe1_0333, 0615, 2026) were found in the genome. Ferredoxin encoding gene TepiRe1_0615 is part of the WL pathway operon; TepiRe1_0333 was found to be reverse-transcribed close to the second *fhs* cluster (described above). Six enzymatic activities were predicted to use ferredoxin as electron transfer protein: a Rnf complex (described below), a pyruvate ferredoxin oxidoreductase (TepiRe1_2369–2371), a carbon monoxide dehydrogenase (part of the WL pathway) and three more potential 2-oxoacid:ferredoxin-dependent oxidoreductases (TepiRe1_2397–2398; 0074–0075, 1413–1415). No evidence for employing cytochromes was found on genome scale. However, genes related to menaquinone biosynthesis are predicted using the MaGe metabolic profile function.

Ferredoxin encoding gene TepiRe1_2026 is part of the recently described electron transport complex Rnf reviewed byBiegel et al. [58], using the redox span between ferredoxin (E` = −500 mV) and NADH (E` = −280 mV) to establish an ion gradient. The genes are organised in the order *rnfCDGEAB* (TepiRe1_2026–2031), as found for many Clostridia and summarised in [58]. A potential [Fe-Fe] hydrogenase gene cluster (TepiRe1_2033–2037) is predicted adjacent to the Rnf cluster, showing similarities to the electron-bifurcating ferredoxin- and NAD^+^-dependent [Fe-Fe] hydrogenase HydABC of *M*. *thermoacetica* and *A*. *woodii* recently characterised by Wang et al. [59] and Schuchmann and Muller [60], respectively, as well as to the [Fe-Fe] hydrogenase of *Thermotoga maritima* [61]. A similar gene cluster has been predicted for the thermophilic SAO *T*. *phaeum*, but no Rnf complex has been found encoded in its genome [28]. Biochemical studies have shown that the purified HydABC complex of *M*. *thermoacetica* and *T*. *maritima* can either evolve or consume hydrogen. There is also strong experimental evidence that hydrogen evolution *in vivo* takes place via HydABC exclusively when growing heterotrophically, reflecting a hydrogen forming rather than consuming function of the complex. A duplication of the [Fe-Fe] hydrogenase (TepiRe1_2699–2701) was found encoded by the genome, similar to the second [Fe-Fe] hydrogenase cluster found in *M*. *thermoacetica* (and *A*. *woodii)*, which were predicted to function as an NADP^+^-reducing [Fe-Fe] hydrogenase based on the similarity to *Desulfovibrio fructosovorans* [59]. Moreover, a putative transhydrogenase catalysing the reversible transversion of NADP^+^ to NADH was found in both *T*. *phaeum* and *T*. *acetatoxydans* (TepiRe1_1871–1873) and might be an important adaptation to the anabolic and catabolic demands of the pathways used under heterotrophic or syntrophic growth and their respective oxidoreductases. The homoacetogens *M*. *thermoacetica*, *A*. *woodii* and *Clostridium ljungdahlii* and their closest relative *T*. *oceani* do not harbour similar genes. Another putative dehydrogenase consisting of at least two subunits (TepiRe1_0818–0819) is only predicted for *T*. *acetatoxydans*.

Interestingly, two ATP synthases are encoded by the genome, but both are predicted to belong to V-type rather than to F-type ATP synthases. V-ATP synthases build up sodium ion or proton gradients at the expense of ATP. The prokaryotic V-ATP synthase consists of nine different subunits: A and B form the peripheral headpiece V_1_ (stator), I and K form the integral membrane complex V_0_ (rotor) and subunits C, D, E, F, G are considered to form the central and peripheral stalk region [62]. The gene order of the first operon is *b(G)*,*C*, *I*, *K*, *F*, *E*, *A*, *B*, *D* (TepiRe1_557–565), as is the case for *T*. *oceani*. The first gene product, designated “b”, shows only low similarity to any function of ATP synthase subunits, but exhibits at least 30% identity to subunit b of F_0_F_1_-ATP synthase of *T*. *maritima* and is considered to be the peripheral paralogue of subunit G in V-type ATP synthases. The second operon shows the gene order *G*, *I*, *K*, *E*, *C*, *F*, *A*, *B*, *D* (TepiRe1_2235–2244) and has been predicted for the closest relative *T*. *oceani* in the same gene order, but has not been found in other sequenced homoacetogens. The first operon has also been predicted in *A*. *woodii* but here the first gene designated *b(G)* is missing. V-ATP synthases can be involved in active transport of metabolites or homeostasis and are assumed not to work in reverse and for synthesis of ATP. However, mechanical modulation experiments performed by [63] suggest conditions under which ATP synthesis might still occur. Moreover, A-ATP synthases found in Archaea function like F-ATP synthases but are structurally rather similar to V-ATP synthases [64]. Thus ATP synthesis activity encoded by the two putative operons described cannot be excluded entirely. On the other hand, in addition to the two V-ATP synthases the genome of *T*. *oceani* harbours a complete F_1_F_0_ ATP synthase gene set. As consequence an electrochemical gradient most likely cannot be used by *T*. *acetatoxydans* to drive ATP synthesis.

### Syntrophic acetate oxidation (SAO)

It has been postulated since the late 1980s that SAO occurs by employing the reverse WL pathway as is the case in sulphate-reducing bacteria, using sulphate as terminal electron acceptor or methanogens coupling acetate oxidation to methanogenesis [9, 50, 65–67]. SAO is thermodynamically unfavourable unless the end product, H_2_ (or formate), is removed immediately. The observed floc formation (Fig. 1) is a morphological trait supporting prompt formate/H_2_ removal. Moreover, the small energy amount gained (*ΔG°´* = −36 kJ/mol) needs to be shared by both partners [20]. The static behaviour of the co-culture even at higher methane production activities shown in Fig. 2 reflects this energy limitation impressively. Genomic evidence of effective communication is described in the sections above. The following section discusses issues related to the SAO pathway.

#### Acetate uptake

In general, the cytoplasma membrane is considered to be permeable to the uncharged form of weak acids such as acetate. However, pH values between 7.0 and 8.5 prevailing in biogas process are not favourable for the uncharged form, which has a *pKs* of 4.76. Thus, *T*. *acetatoxydans* might metabolically profit from process-related acetate accumulation and pH drops due to the increased passive influx. Active monocarboxylic acid uptake transporter ActP (MctC) belonging to the SSS family (sodium solute symporter/carrier 5 family) has been described for *Corynebacterium glutamicum* (MctC), channelling pyruvate, acetate and propionate [68], *Rhizobium leguminosarum* (MctP), importing pyruvate, lactate and alanine [69], and *Escherichia coli* (ActP), taking up only acetate [70]. In *T*. *acetatoxydans*, a separately located CDS (TepiRe1_0838) is predicted to belong to the same sodium solute transporter family, sharing 39% similarity with MctC (S6 Table).

Digester studies carried out in our laboratory showed a distinct correlation between increasing acetate concentrations and increasing abundance of *T*. *acetatoxydans*, which might point to passive rather than active uptake (unpublished). It has also been shown by [57] that pH values below 6.8 turn the potential FocA transporter (section “Acetogenesis”) into an active formate and acetate import system using the proton gradient. Both ways might be utilised by *T*. *acetatoxydans*.

#### Acetate activation

Three ways have been described for activation of acetate: The first is a reversion of the activity of phosphate acetyl transferase and acetate kinase (TepiRe1_1558–1559), requiring an ATP as has been described for sulphate reducers [71] and is well studied in aceticlastic methanogens [72, 73]. The second mechanism is performed by an AMP-forming acetyl-CoA synthetase (EC 6.2.11), as found in enteric bacteria, and is considered a high affinity pathway when the acetate concentration becomes low [74]. However, no similar gene seems to be encoded by the genome of *T*. *acetatoxydans*. The third way involves the transfer of the CoA moiety preserving the energy as a thioester bond [75]. This is the case *e*.*g*. in *Clostridium acetobutylicum*, where a CoA transferase (EC 2.3.19) transfers the CoA moiety from one carboxylic acid to another [75]. This transferase has been shown to have broad substrate specificity, including acetate, formate, propionate, butyrate, crotonate and valerate. A CoA transferase is predicted to be part of a putative operon consisting of the ORFs (TepiRe1_1963–1971). If acetate needs to be taken up together with another monocarboxylic acid, the acetate would be oxidised completely and the other acid would be released modified into the medium. So far, we have not observed any evidence of the latter option, but in-depth experiments have not yet been conducted.

#### Energy conservation during SAO

Two activities of the oxidative WL pathway (formate dehydrogenase, CO dehydrogenase) are exergonic [76], and might generate an electrochemical gradient by coupling the oxidation of acetate via ferredoxins to the Rnf complex and potential [Fe-Fe] hydrogenases mentioned above. Due to the lack of formate dehydrogenase, the oxidation of acetate by reversing the WL pathway results in formate, CO_2_ and hydrogen. As consequences, i) the methanogenic partner must be able to consume both formate and H_2_, as has been shown for the isolated methanogenic partner *Methanoculleus* sp. MAB2 [18]; and ii) even less energy than calculated by [20] becomes available for *T*. *acetatoxydans*. Moreover, the energy gained most likely cannot be converted into ATP due to the lack of F_1_F_0_ ATP synthase activity and the ATP obtained by substrate-level phosphorylation during formyltetrahydrofolate synthase activity is needed for the activation of acetate (section “Acetate activation”). Consequently, net production of ATP remains zero. Although the energy saved in an electrochemical gradient can be used directly for *e*.*g*. solute uptake, secretion, homeostatis and chemotaxis, surviving even statically seems inconceivable.

Therefore, we suggest that another pathway might be used by *T*. *acetatoxydans* rather than the WL pathway. In the late 1980s, an oxidative tricarboxylic acid cycle was suggested for the sulphate-reducing bacteria *Desulfobacter postgatei* and *Desulfobacter hydrogenophilus* [77, 78]. Based on enzyme activity studies, the authors suggested acetate activation via CoA-transfer from succinyl-CoA. Succinyl-CoA in turn becomes regenerated by the activity of α-ketoglutarate:ferredoxin oxidoreductase. Acetyl-CoA reacts with oxalacetate to form citrate and regains one ATP net. Citrate is further oxidised back to α-ketoglutarate. According to our genome-scale analysis, all the enzyme activities needed appear to be encoded in the *T*. *acetatoxydans* genome (see section “substrate utilization”) and represent a potential alternative to the WL pathway. Rnf complex and bifurcating [Fe-Fe] hydrogenases might further link the pathway to energy-conserving reactions by regenerating the eight reduction equivalents produced and providing energy for proton gradient-consuming cell activities. Interestingly, the predicted formate/nitrite transporter (TepiRe1_2032) mentioned above in section on acetogenesis was found encoded between the *rnf* (TepiRe1_2026–2031) and the hydrogen-forming [Fe-Fe] hydrogenase gene clusters (TepiRe1_2033–2037). Thus, a function in both formate and acetate export using the proton gradient produced might pull either pathway, SAO or acetate formation. Furthermore, Thauer and coworkers [59] found evidence of a biochemical link between increased H_2_ evolution of the [Fe-Fe] hydrogenase HydABC of *M*. *thermoacetica* and increasing pH. Thus, a slightly alkaline pH as the expected consequence of the ammonia pressure prevailing in protein-rich AD processes might be beneficial for the thermodynamics of the SAO pathway. The thermophilic, methanol-degrading bacterium *T*. *lettingae* has been shown to possess SAOB ability, but there is no evidence that it uses the WL pathway, based on substrate utilisation patterns [27]. In contrast, the thermophilic SAOB *T*. *phaeum* lacks Rnf complex but harbours both formate dehydrogenase and F_1_F_0_-ATP synthase and might employ the WL pathway, as supported by the enzyme activities measured [67].

#### Syntrophy versus free-living

The metabolic behaviour of *T*. *acetatoxydans* in non-SAO-dominated processes is difficult to predict. Does the organism relinquish the syntrophy and instead live independently of the methanogenic partner? The genome analysis presented here revealed all the essential functions of a “free”-living style. Nevertheless, the low heterotrophic growth rates achieved and the moderate substrate spectrum [25] do not suggest high competitiveness in complex environments. Moreover, no net ATP can be gained by expressing the WL pathway, due to its predicted restriction in anaerobic respiration (see the “Energy conservation” section). On the other hand, forming a syntrophic interaction and establishing acetate oxidation is, at least in defined co-cultures, time-consuming and might even be an energy-depleting process. In *Acetobacterium woodii*, when growing on fructose in co-culture with a hydrogenotrophic methanogen, the electron flow is directed towards hydrogenases, evolving H_2_ directly consumed by the methanogenic partner and bypassing the W-L pathway [79]. The same mechanism can potentially be used by *T*. *acetatoxydans* as the genome harbors similar sets of [Fe-Fe] hydrogenases, as found in the genome of *A*. *woodii* (see the “Energy conservation” section). Due to the formate dependency of the WL pathway, such bypassing might broaden the substrate spectrum and subsequently improve competitiveness. Thus, the syntrophic lifestyle might be preferred by *T*. *acetatoxydans*, enabling the bacteria to react to the nutrient situation more efficiently once syntrophy has been established. Under high ammonium conditions, it might have a preference for oxidising acetate as the most available non-competitive substrate. However, the prevailing ammonium concentration might steer the rise and fall of this syntrophic niche, irrespective of the substrate utilized syntrophically.

## Conclusions

Several genomic traits were found in the genome of *T*. *acetatoxydans* which might be indicative of adaptation to ammonia-stressed AD processes, clearly distinguishing this organism from other acetogenic model bacteria sequenced and also explaining its rareness in other habitats. The lack of formate dehydrogenase and F_1_F_0_ ATP synthase creates an enigma regarding energy conservation, especially ATP generation. It can be concluded that mesophilic SAOB likely use different metabolic strategies for acetate oxidation. The use of the Wood-Ljungdahl pathway might not always be the catabolic solution as previously postulated, even if encoded by the genome.

The genomic picture presented here should be considered a reflection of the potential physiological capabilities of *T*. *acetatoxydans*, while the actual phenotypic characteristics of the syntrophy need to be identified in future transcriptome and proteome studies. However this genome-guided analysis provides essential information needed for steering, optimising and controlling SAO biogas processes, reveals possible threats to SAOB with consequences for the biogas process and provides a valuable platform for further ‘omics’ applications.

## Materials and Methods

### Cultivation

As the growth medium for *T*. *acetatoxydans*, bicarbonate-buffered basal medium (BM) was prepared by mixing solutions A-I as described by Zehnder et al. [65] with some modifications as described by Westerholm et al. [24]. In brief, 15 mL of solution A, 15 mL of solution B, 1 mL of solution F, 5 mL of solution I and 0.2 g L ^−1^ yeast extract were added to 1 L of distilled water and boiled for approximately 20 min to a final volume of 900 mL. The medium was dispensed into 500 mL bottles under flushing with N_2_/CO_2_ (80/20 v/v). The bottles were sealed with butyl rubber stoppers and aluminium lids and autoclaved for 20 min at 121°C. Subsequently, mixture C1, containing 1 mL of trace metal solution E, 1 mL of vitamin solution G, 12.5 mL of solution C and 34.5 mL distilled water, and mixture C2, containing 49 mL of solution D, 1 mL of solution H and 0.5 g of cysteine-HC1, were prepared separately and sterile-filtered (0.2 μm) into closed autoclaved vials filled with N_2._. Next, 10 mL portions of each mixture were transferred by syringe to bottles containing 180 mL medium. Cultures of *T*. *acetatoxydans* were cultivated on 10 mM glucose in the dark at 37°C without shaking. In order to confirm syntrophic acetate-oxidising ability, the SAOB were inoculated to cultures with high cell density of the hydrogen-utilising methanogen *Methanoculleus* sp. strain MAB2 [18]. Methane production was determined by gas chromatography (GC) according to Westerholm et al. [24] and growth was quantitatively monitored by qPCR as described elsewhere [22, 37].

### Genome Assembly

DNA was extracted using the Blood & Tissue Kit from Qiagen (Hilden, Germany) from cells cultivated as described above. The *T*. *acetatoxydans* genome (HF563609) was sequenced and *de novo* assembly was performed as described by Manzoor *et al*. [29]. Low-quality and short reads were filtered using the *Sickle* tool [80]. In addition, the filtered reads were mapped to the published *T*. *acetatoxydans* (NC_015519) genome using the Mimicking Intelligent Read Assembly (MIRA) assembler [81]. Of the total reads, 88% could be mapped to the reference genome with 156-fold sequencing coverage across the entire genome by producing the consensus sequence of size 2,761,826 bp. Subsequently, gaps among the *de novo* assembled scaffolds were filled by combining the *de novo* assembly with the mapping assembly draft using the following three steps. Sorted scaffolds were concatenated by aligning to the mapping assembly draft using the genome alignment *Mauve* tool [82] and assembled into large scaffolds as follows: (i) Two adjacent scaffolds that overlapped were merged into one larger scaffold; (ii) if a subsequence was inserted in the mapping assembly draft between two adjacent scaffolds, such scaffolds and the inserted subsequence were concatenated into one scaffold; (iii) by using these steps, one large scaffold in the form of a final draft sequence with size 2,761,252 was constructed from 96 scaffolds. This final draft sequence size is slightly higher than that reported in our genome announcement [29].

### Genome Annotation

The assembled genome was annotated by using Magnifying Genomes (MaGe) [83] a bacterial genome annotation system. Different algorithms were used to predict the putative CDSs such as: i) *Glimmer* (Gene Locator and Interpolated Markov ModelER) [84], ii) *AMIGene* (Annotation of Microbial Genes) application [85] and iii) *Prodigal* (PROkaryotic DYnamic programming Gene-finding Algorithm) [86]. tRNAs were predicted using the *tRNAScanSE* tool [87]. Predicted CDSs were translated and used to search the National Center for Biotechnology Information (NCBI) non-redundant database, UniProt, TIGRFam, Pfam, PRIAM, KEGG, COG, and InterPro databases using Basic local alignment search tool for proteins (BLASTP). SignalIP neural network software [88] was used for signal peptide prediction. Transmembrane regions were predicted through the TMHMM server, which analyses the physical constraints of both soluble and membrane-based sequences with up to 90% accuracy [89]. Predicted genes and operons were also subjected to manual analysis using the MaGe web-based platform, in order to assess and correct genes and operons of interest.

## Supporting Information

S1 Table Number of genes associated with the general COG functional categories. (DOCX)

S2 Table Statistics on the three prophage regions identified in the genome of Tepidanaerobacter acetatoxydans strain Re1. (DOCX)

S3 Table Putative ABC components identified in the genome of *Tepidanaerobacter acetatoxydans* strain Re1. (DOCX)

S4 Table Putative TRAP transporter-encoding genes identified in the genome of *Tepidanaerobacter acetatoxydans* strain Re1. (DOCX)

S5 Table Putative antiporter genes identified in the genome of *Tepidanaerobacter acetatoxydans* strain Re1. (DOCX)

S6 Table Putative symporter genes identified in the genome of *Tepidanaerobacter acetatoxydans* strain Re1. (DOCX)

S7 Table Putative PTS components identified in the genome of *Tepidanaerobacter acetatoxydans* strain Re1. (DOCX)
