# Supplementary material for: Genome-Guided Analysis of Physiological Capacities of Tepidanaerobacter acetatoxydans Provides Insights into Environmental Adaptations and Syntrophic Acetate Oxidation
Source: PLoS One. 2015 Mar 26;10(3):e0121237. doi: 10.1371/journal.pone.0121237 (PMC4374699; doi:10.1371/journal.pone.0121237)
Supplement: S1 Table — (DOCX) [file pone.0121237.s001.docx]

| **Code** | **Value** | **% age** | **Description** |
| --- | --- | --- | --- |
| J | 142 | 5.3464 | Translation, ribosomal structure and biogenesis |
| K | 194 | 7.3042 | Transcription |
| L | 170 | 6.4006 | Replication, recombination and repair |
| B | 1 | 0.0377 | Chromatin structure and dynamics |
| D | 52 | 1.9578 | Cell cycle control, cell division, chromosome partitioning |
| V | 48 | 1.8072 | Defence mechanisms |
| T | 137 | 5.1581 | Signal transduction mechanisms |
| M | 113 | 4.2545 | Cell wall/membrane/envelope biogenesis |
| N | 69 | 2.5979 | Cell motility |
| U | 50 | 1.8825 | Intracellular trafficking, secretion and vesicular transport |
| O | 66 | 2.4849 | Posttranslational modification, protein turnover, chaperones |
| C | 157 | 5.9111 | Energy production and conversion |
| G | 242 | 9.1114 | Carbohydrate transport and metabolism |
| E | 287 | 10.8057 | Amino acid transport and metabolism |
| F | 57 | 2.1461 | Nucleotide transport and metabolism |
| H | 85 | 3.2003 | Coenzyme transport and metabolism |
| I | 45 | 1.6943 | Lipid transport and metabolism |
| P | 103 | 3.8780 | Inorganic ion transport and metabolism |
| Q | 32 | 1.2048 | Secondary metabolites biosynthesis, transport and catabolism |
| R | 292 | 10.9940 | General function prediction only |
| S | 203 | 7.6431 | Function unknown |
|  | 498 | 18.75 | Not in COGs |
