# Supplementary material for: Genome-Guided Analysis of Physiological Capacities of Tepidanaerobacter acetatoxydans Provides Insights into Environmental Adaptations and Syntrophic Acetate Oxidation
Source: PLoS One. 2015 Mar 26;10(3):e0121237. doi: 10.1371/journal.pone.0121237 (PMC4374699; doi:10.1371/journal.pone.0121237)
Supplement: S2 Table — (DOCX) [file pone.0121237.s002.docx]

| Start | End | Length  Kb | Total  proteins | Phage  proteins | Hypothetical proteins | Bacteriall  proteins | tRNA | Status |
| --- | --- | --- | --- | --- | --- | --- | --- | --- |
| 1,075,607 | 1,111,233 | 35.6 | 51 | 28 | 22 | 1 | 1 | Complete |
| 2,500,103 | 2,538,024 | 37.9 | 49 | 34 | 13 | 2 | 0 | Complete |
| 1,495,922 | 1,521,124 | 25.2 | 29 | 15 | 4 | 10 | 2 | Incomplete |
