# Supplementary material for: Genome-Guided Analysis of Physiological Capacities of Tepidanaerobacter acetatoxydans Provides Insights into Environmental Adaptations and Syntrophic Acetate Oxidation
Source: PLoS One. 2015 Mar 26;10(3):e0121237. doi: 10.1371/journal.pone.0121237 (PMC4374699; doi:10.1371/journal.pone.0121237)
Supplement: S3 Table — (DOCX) [file pone.0121237.s003.docx]

| **Label** | **Begin** | **End** | **Length** | **Product** |  |
| --- | --- | --- | --- | --- | --- |
| TepiRe1_0041 | 39160 | 40809 | 1650 | ABC-type transporter, periplasmic subunit |  |
| TepiRe1_0042 | 40910 | 41830 | 921 | Membrane component of ABC superfamily | |
| TepiRe1_0044 | 42808 | 43812 | 1005 | Oligopeptide ABC transporter (ABC subunit) | |
| TepiRe1_0045 | 43754 | 44785 | 1032 | oligopeptide ABC transporter (ABC subunit) | |
| TepiRe1_0105 | 109648 | 110562 | 915 | ABC-type metal ion transporter, periplasmic subunit | |
| TepiRe1_0368 | 358986 | 360635 | 1650 | ABC-type transporter, periplasmic subunit | |
| TepiRe1_0369 | 360632 | 361600 | 969 | ABC-type transporter, integral membrane subunit | |
| TepiRe1_0370 | 361613 | 362443 | 831 | ABC-type transporter, integral membrane subunit | |
| TepiRe1_0371 | 362488 | 364182 | 1695 | Oligopeptide/dipeptide ABC transporter, ATPase subunit | |
| TepiRe1_0428 | 418946 | 419650 | 705 | ABC superfamily | |
| TepiRe1_0429 | 419644 | 420426 | 783 | ATP-binding component of ABC superfamily | |
| TepiRe1_0430 | 420430 | 421293 | 864 | ABC-type transporter, integral membrane subunit | |
| TepiRe1_0431 | 421305 | 422174 | 870 | ABC superfamily | |
| TepiRe1_0470 | 460496 | 461548 | 1053 | ABC transporter permease protein | |
| TepiRe1_0471 | 461536 | 462333 | 798 | ABC transporter ATP-binding protein | |
| TepiRe1_0472 | 462384 | 463505 | 1122 | ABC-type transporter, periplasmic subunit | |
| TepiRe1_0489 | 478120 | 479754 | 1635 | ABC-type transporter, periplasmic subunit | |
| TepiRe1_0490 | 480032 | 480997 | 966 | Oligopeptide ABC transporter (ATP-binding protein) | |
| TepiRe1_0491 | 480969 | 481979 | 1011 | oligopeptide ABC transporter (ATP binding protein) | |
| TepiRe1_0706 | 704487 | 705986 | 1500 | ABC-type transporter, integral membrane subunit | |
| TepiRe1_0707 | 705992 | 706996 | 1005 | membrane component of ABC superfamily | |
| TepiRe1_0808 | 811072 | 812139 | 1068 | ABC superfamily | |
| TepiRe1_0809 | 812219 | 813730 | 1512 | ABC superfamily: ATP-binding components | |
| TepiRe1_0810 | 813764 | 814777 | 1014 | Methyl-galactoside transporter subunit; membrane component of ABC | |
| TepiRe1_0845 | 853769 | 855622 | 1854 | ABC-type transporter, periplasmic subunit | |
| TepiRe1_0846 | 855761 | 856705 | 945 | ABC-type transporter, integral membrane subunit | |
| TepiRe1_0847 | 856705 | 857673 | 969 | ABC-type transporter, integral membrane subunit | |
| TepiRe1_0848 | 857685 | 858734 | 1050 | Oligopeptide ABC transporter (ATP-binding protein) | |
| TepiRe1_0849 | 858727 | 859713 | 987 | Oligopeptide ABC transporter (ATP-binding protein) | |
| TepiRe1_0942 | 945845 | 946582 | 738 | ABC-2 type transporter | |
| TepiRe1_0961 | 962346 | 963101 | 756 | ABC-2 type transporter | |
| TepiRe1_0983 | 978191 | 979687 | 1497 | ABC transporter related protein | |
| TepiRe1_1060 | 1028717 | 1029448 | 732 | Putative ABC transporter, permease protein | |
| TepiRe1_1078 | 1046359 | 1047225 | 867 | Phosphate ABC transporter, inner membrane subunit PstC | |
| TepiRe1_1079 | 1047222 | 1048058 | 837 | Phosphate ABC transporter, inner membrane subunit PstA | |
| TepiRe1_1082 | 1050651 | 1051406 | 756 | Phosphate ABC transporter (ATP-binding protein) | |
| TepiRe1_1090 | 1057971 | 1059395 | 1425 | ABC-type uncharacterized transport system | |
| TepiRe1_1091 | 1059409 | 1060119 | 711 | ABC-2 type transporter | |
| TepiRe1_1096 | 1064316 | 1065269 | 954 | ABC-type transporter, periplasmic subunit | |
| TepiRe1_1699 | 1614029 | 1615054 | 1026 | ABC transporter substrate binding protein leucine/isoleucine/valine | |
| TepiRe1_1883 | 1799335 | 1800048 | 714 | ABC superfamily leucine/isoleucine/valine transporter subunit | |
| TepiRe1_1884 | 1800051 | 1800842 | 792 | ABC superfamily | |
| TepiRe1_1885 | 1800848 | 1801822 | 975 | ABC-type transporter, integral membrane subunit | |
| TepiRe1_1886 | 1801834 | 1802721 | 888 | ABC-type transporter, integral membrane subunit | |
| TepiRe1_1929 | 1847056 | 1848237 | 1182 | ABC-type antimicrobial peptide transport system, permease component | |
| TepiRe1_1950 | 1870050 | 1870760 | 711 | leucine/isoleucine/valine transporter subunit ; ATP-binding component | |
| TepiRe1_1951 | 1870762 | 1871556 | 795 | Leucine/isoleucine/valine transporter subunit ; ATP-binding component | |
| TepiRe1_1952 | 1871558 | 1872532 | 975 | ABC-type transporter, integral membrane subunit | |
| TepiRe1_1953 | 1872551 | 1873420 | 870 | ABC-type transporter, integral membrane subunit | |
| TepiRe1_1979 | 1897120 | 1898133 | 1014 | Methyl-galactoside transporter subunit ; membrane component of ABC | |
| TepiRe1_1980 | 1898167 | 1899732 | 1566 | ATP-binding components | |
| TepiRe1_1981 | 1899757 | 1900824 | 1068 | Methyl-galactoside transporter subunit; periplasmic-binding component | |
| TepiRe1_1982 | 1900988 | 1902064 | 1077 | D-xylose transporter subunit; periplasmic-binding component of ABC | |
| TepiRe1_2045 | 1969416 | 1970390 | 975 | Oligopeptide ABC transporter (ATP-binding protein) | |
| TepiRe1_2046 | 1970391 | 1971377 | 987 | Oligopeptide ABC transporter (ATP-binding protein) | |
| TepiRe1_2047 | 1971389 | 1972306 | 918 | Dipeptide transporter; membrane component of ABC superfamily | |
| TepiRe1_2048 | 1972321 | 1973247 | 927 | Putative peptide transporter permease subunit: membrane component of | |
| TepiRe1_2049 | 1973335 | 1974993 | 1659 | ABC-type transporter, periplasmic subunit | |
| TepiRe1_2116 | 2043703 | 2044443 | 741 | Histidine/lysine/arginine/ornithine transporter subunit; ATP-binding component | |
| TepiRe1_2117 | 2044505 | 2045233 | 729 | Polar amino acid ABC transporter, inner membrane subunit | |
| TepiRe1_2119 | 2045415 | 2046269 | 855 | ABC-type transporter, periplasmic subunit | |
| TepiRe1_2151 | 2088199 | 2089113 | 915 | ABC transporter permease protein | |
| TepiRe1_2152 | 2089114 | 2090232 | 1119 | ABC-type transporter, integral membrane subunit | |
| TepiRe1_2153 | 2090229 | 2091755 | 1527 | ABC transporter ATP-binding protein | |
| TepiRe1_2211 | 2147177 | 2148175 | 999 | ABC-type transporter, integral membrane subunit | |
| TepiRe1_2212 | 2148187 | 2149686 | 1500 | subunits of ABC superfamily: ATP-binding components | |
| TepiRe1_2253 | 2186894 | 2187727 | 834 | ABC-type transporter, integral membrane subunit | |
| TepiRe1_2255 | 2187729 | 2188610 | 882 | ABC-type transporter, integral membrane subunit | |
| TepiRe1_2517 | 2448155 | 2448829 | 675 | Putative transporter subunit: permease component of ABC superfamily | |
| TepiRe1_2518 | 2448840 | 2449634 | 795 | Glutamine ABC transporter (ATP-binding protein) | |
| TepiRe1_2519 | 2449624 | 2450295 | 672 | Polar amino acid ABC transporter, inner membrane subunit | |
| TepiRe1_2521 | 2451319 | 2452137 | 819 | ABC-type transporter, periplasmic subunit family 3 | |
| TepiRe1_2538 | 2469798 | 2470763 | 966 | ABC-type glycine betaine transport, periplasmic subunit | |
| TepiRe1_2539 | 2470797 | 2471645 | 849 | ABC-type transporter, integral membrane subunit | |
| TepiRe1_2540 | 2471670 | 2472848 | 1179 | Glycine betaine ABC transporter (ATP-binding protein) | |
| TepiRe1_2709 | 2630357 | 2631355 | 999 | ABC-type transport, permease protein | |
| TepiRe1_2799 | 2717320 | 2718069 | 750 | glutamine transporter subunit ; ATP-binding component of ABC | |
| TepiRe1_2800 | 2718171 | 2718725 | 555 | Polar amino acid ABC transporter, inner membrane subunit | |
| TepiRe1_2801 | 2718803 | 2719657 | 855 | ABC-type transporter, periplasmic subunit family 3 | |
