# Supplementary material for: Genome-Guided Analysis of Physiological Capacities of Tepidanaerobacter acetatoxydans Provides Insights into Environmental Adaptations and Syntrophic Acetate Oxidation
Source: PLoS One. 2015 Mar 26;10(3):e0121237. doi: 10.1371/journal.pone.0121237 (PMC4374699; doi:10.1371/journal.pone.0121237)
Supplement: S4 Table — (DOCX) [file pone.0121237.s004.docx]

| Label | Begin | End | Length | Product |
| --- | --- | --- | --- | --- |
| TepiRe1_0144 | 151589 | 152620 | 1032 | TRAP transporter subunits |
| TepiRe1_0146 | 153150 | 154430 | 1281 | TRAP transporter subunits |
| TepiRe1_0867 | 877316 | 879262 | 1947 | TRAP transporter subunits |
| TepiRe1_0870 | 879815 | 880813 | 999 | TRAP transporter subunits |
| TepiRe1_1708 | 1625485 | 1626774 | 1290 | TRAP transporter subunits |
| TepiRe1_1710 | 1627328 | 1628368 | 1041 | TRAP transporter subunits |
| TepiRe1_1970 | 1889715 | 1891004 | 1290 | TRAP transporter subunits |
| TepiRe1_1972 | 1891571 | 1892596 | 1026 | TRAP transporter subunits |
| TepiRe1_2107 | 2034998 | 2036272 | 1275 | TRAP transporter subunits |
| TepiRe1_2109 | 2036853 | 2037911 | 1059 | TRAP transporter subunits |
| TepiRe1_2439 | 2371762 | 2373645 | 1884 | TRAP transporter subunits |
