# Supplementary material for: Genome-Guided Analysis of Physiological Capacities of Tepidanaerobacter acetatoxydans Provides Insights into Environmental Adaptations and Syntrophic Acetate Oxidation
Source: PLoS One. 2015 Mar 26;10(3):e0121237. doi: 10.1371/journal.pone.0121237 (PMC4374699; doi:10.1371/journal.pone.0121237)
Supplement: S5 Table — (DOCX) [file pone.0121237.s005.docx]

| Label | Begin | End | Length | Product |
| --- | --- | --- | --- | --- |
| TepiRe1_0140 | 146204 | 147232 | 1029 | Na+/H+ antiporter |
| TepiRe1_0933 | 935505 | 936800 | 1296 | Na+/H+ antiporter |
| TepiRe1_1094 | 1061720 | 1063099 | 1380 | Na+/H+ antiporter |
| TepiRe1_2020 | 1945015 | 1946409 | 1395 | Na+/H+ antiporter |
