# Supplementary material for: Genome-Guided Analysis of Physiological Capacities of Tepidanaerobacter acetatoxydans Provides Insights into Environmental Adaptations and Syntrophic Acetate Oxidation
Source: PLoS One. 2015 Mar 26;10(3):e0121237. doi: 10.1371/journal.pone.0121237 (PMC4374699; doi:10.1371/journal.pone.0121237)
Supplement: S6 Table — (DOCX) [file pone.0121237.s006.docx]

| Label | Begin | End | Length | Product |
| --- | --- | --- | --- | --- |
| TepiRe1_0019 | 19344 | 20780 | 1437 | a+/solute symporter |
| TepiRe1_0327 | 315279 | 316676 | 1398 | a+/solute symporter |
| TepiRe1_0569 | 557480 | 558712 | 1233 | a+/solute symporter |
| TepiRe1_0838 | 845558 | 846955 | 1398 | a+/solute symporter |
| TepiRe1_1787 | 1707260 | 1708618 | 1359 | a+/solute symporter |
| TepiRe1_2001 | 1920051 | 1921541 | 1491 | a+/solute symporter |
| TepiRe1_2072 | 1995826 | 1997055 | 1230 | a+/solute symporter |
| TepiRe1_2745 | 2452783 | 2454144 | 1362 | a+/solute symporter |
