# Supplementary material for: Genome-Guided Analysis of Physiological Capacities of Tepidanaerobacter acetatoxydans Provides Insights into Environmental Adaptations and Syntrophic Acetate Oxidation
Source: PLoS One. 2015 Mar 26;10(3):e0121237. doi: 10.1371/journal.pone.0121237 (PMC4374699; doi:10.1371/journal.pone.0121237)
Supplement: S7 Table — (DOCX) [file pone.0121237.s007.docx]

| **Label** | **Begin** | **End** | **Length** | **Product** | |  |
| --- | --- | --- | --- | --- | --- | --- |
| TepiRe1_0054 | 56561 | 57877 | 1317 | PTS system lactose/cellobiose-specific transporter subunit IIB |  |  |
| TepiRe1_0058 | 60783 | 62657 | 1875 | PTS system lactose/cellobiose family transporter subunit IIC | | |
| TepiRe1_0300 | 287832 | 288659 | 828 | PTS lactose/cellobiose-specific subunit IIA | | |
| TepiRe1_0301 | 288652 | 289413 | 762 | PTS system mannose/fructose/sorbose family transporter subunit IID | | |
| TepiRe1_0302 | 289426 | 289902 | 477 | PTS sorbose-specific transporter subunit IIC | | |
| TepiRe1_0303 | 289902 | 290309 | 408 | PTS system sorbose subfamily transporter subunit IIB | | |
| TepiRe1_0671 | 665385 | 668390 | 3006 | PTS system fructose subfamily transporter subunit IIA | | |
| TepiRe1_0672 | 668532 | 669005 | 474 | PTS system transporter subunit IIC | | |
| TepiRe1_0674 | 669345 | 670694 | 1350 | PTS system lactose/cellobiose-specific transporter subunit IIB | | |
| TepiRe1_0694 | 689608 | 690135 | 528 | PTS system galactitol-specific transporter subunit IIC | | |
| TepiRe1_0695 | 690152 | 691147 | 996 | PTS system glucitol/sorbitol-specific transporter subunit IIC | | |
| TepiRe1_0696 | 691226 | 691591 | 366 | PTS system glucitol/sorbitol-specific transporter subunit IIA | | |
| TepiRe1_1085 | 1053133 | 1053501 | 369 | PTS system glucitol/sorbitol-specific transporter subunit IIA | | |
| TepiRe1_1088 | 1055214 | 1056935 | 1722 | PTS system lactose/cellobiose family transporter subunit IIC | | |
| TepiRe1_1760 | 1674392 | 1675747 | 1356 | PTS system lactose/cellobiose-specific transporter subunit IIB | | |
| TepiRe1_1761 | 1675887 | 1676186 | 300 | PTS lactose/cellobiose-specific IIA subunit | | |
| TepiRe1_1762 | 1676239 | 1676568 | 330 | PTS system lactose/cellobiose family transporter subunit IIC | | |
| TepiRe1_1767 | 1681607 | 1682923 | 1317 | PTS lactose/cellobiose-specific subunit IIA | | |
| TepiRe1_1769 | 1684533 | 1684862 | 330 | PTS system sorbose subfamily transporter subunit IIB | | |
| TepiRe1_1770 | 1684978 | 1687677 | 2700 | PTS system mannose/fructose/sorbose family transporter subunit IID | | |
| TepiRe1_1826 | 1751074 | 1751571 | 498 | PTS system fructose subfamily transporter subunit IIA | | |
| TepiRe1_1828 | 1751887 | 1753545 | 1659 | PTS system sorbose subfamily transporter subunit IIB | | |
| TepiRe1_1830 | 1755088 | 1755546 | 459 | PTS system fructose subfamily transporter subunit IIA | | |
| TepiRe1_1831 | 1755543 | 1756016 | 474 | PTS system fructose subfamily transporter subunit IIA | | |
| TepiRe1_1832 | 1756029 | 1756448 | 420 | PTS system mannose/fructose/sorbose family transporter subunit IID | | |
| TepiRe1_1833 | 1756663 | 1759509 | 2847 | PTS sorbose-specific transporter subunit IIC | | |
| TepiRe1_1863 | 1779605 | 1780507 | 903 | PTS system sorbose subfamily transporter subunit IIB | | |
| TepiRe1_1865 | 1781683 | 1782072 | 390 | PTS system glucose subfamily transporter subunit IIA | | |
| TepiRe1_1866 | 1782149 | 1783015 | 867 | PTS system N-acetyl glucosamine-specific transporter subunit IIBC | | |
| TepiRe1_1867 | 1783019 | 1783801 | 783 | PTS system glucitol/sorbitol-specific transporter subunit IIA | | |
| TepiRe1_1868 | 1783822 | 1784313 | 492 | PTS system sorbitol-specific transporter subunit IIC | | |
| TepiRe1_1875 | 1790809 | 1791315 | 507 | PTS system cellobiose-specific transporter subunit IIC | | |
| TepiRe1_1877 | 1792325 | 1793701 | 1377 | PTS system lactose/cellobiose-specific transporter subunit IIB | | |
| TepiRe1_1995 | 1915740 | 1916156 | 417 | PTS lactose/cellobiose-specific IIA subunit | | |
| TepiRe1_1998 | 1917284 | 1918255 | 972 | PTS system ascorbate-specific transporter subunit IIC | | |
| TepiRe1_1999 | 1918292 | 1918828 | 537 | PTS system lactose/cellobiose-specific transporter subunit IIB | | |
| TepiRe1_2009 | 1929854 | 1930180 | 327 | PTS system lactose/cellobiose-specific transporter subunit IIB | | |
| TepiRe1_2011 | 1931267 | 1934002 | 2736 | PTS system lactose/cellobiose family transporter subunit IIC | | |
| TepiRe1_2174 | 2109071 | 2110372 | 1302 | PTS lactose/cellobiose-specific subunit IIA | | |
| TepiRe1_2176 | 2110709 | 2112844 | 2136 | PTS lactose/cellobiose-specific subunit IIA | | |
| TepiRe1_2268 | 2198719 | 2199159 | 441 | PTS system lactose/cellobiose family transporter subunit IIC | | |
| TepiRe1_2270 | 2199912 | 2201327 | 1416 | PTS system lactose/cellobiose-specific transporter subunit IIB | | |
| TepiRe1_2357 | 2291449 | 2291769 | 321 | PTS system lactose/cellobiose family transporter subunit IIC | | |
| TepiRe1_2358 | 2291804 | 2293075 | 1272 | PTS system lactose/cellobiose-specific transporter subunit IIB | | |
| TepiRe1_2389 | 2323535 | 2324530 | 996 | PTS lactose/cellobiose-specific IIA subunit | | |
| TepiRe1_2412 | 2348163 | 2348483 | 321 | PTS system fructose subfamily transporter subunit IIA | | |
| TepiRe1_2417 | 2351286 | 2352632 | 1347 | PTS system sorbose subfamily transporter subunit IIB | | |
| TepRe1_2737 | 2661503 | 2662258 | 756 | PTS sorbose-specific transporter subunit IIC | | |
| TepRe1_2738 | 2662248 | 2663078 | 831 | PTS system mannose/fructose/sorbose family transporter subunit IID | | |
| TepRe1_2775 | 2698186 | 2699565 | 1380 | PTS system fructose subfamily transporter subunit IIC | | |
